# What is in a name and other peeves

**DOI:** 10.1111/1753-0407.70017

**Published:** 2024-09-27

**Authors:** Zachary Bloomgarden

**Affiliations:** ^1^ Department of Medicine, Division of Endocrinology Diabetes and Bone Disease, Icahn School of Medicine New York USA

“What's in a name? That which we call a rose, by any other word would smell as sweet.”

William Shakespeare, Romeo and Juliet (Act 2, Scene 2)

A number of submissions to the Journal of Diabetes appear to share similar flaws, and it might be of interest to potential authors to understand some of the characteristics which lead the Editors to look unfavorably on such.

**FIGURE 1:**
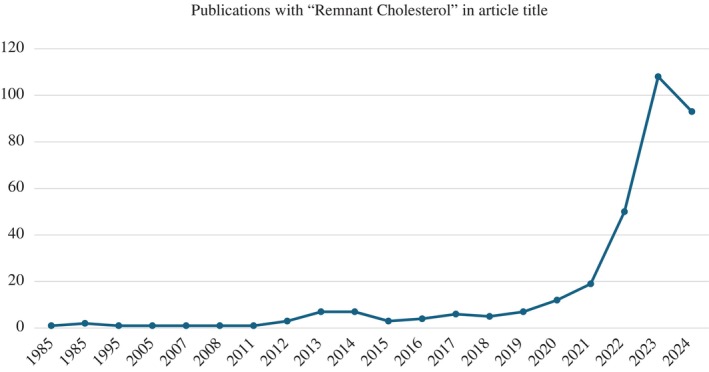
Publications with “Remnant Cholesterol” in article title. Downloaded from https://pubmed.ncbi.nlm.nih.gov/August 4, 2024.

We have lately noticed quite a few submissions pertaining to associations of “remnant cholesterol” (RC) with various diabetes‐related conditions and complications. A PubMed literature search suggests that this is not only true for our Journal, but also has led to many recent publications (Figure 1). Does this reflect some new knowledge of the nature of atherogenic lipoproteins?

Such manuscripts calculate RC as (total cholesterol [TC]—high‐density lipoprotein cholesterol [HDL‐C]—low‐density lipoprotein cholesterol [LDL‐C]). However, most of these use the Friedewald equation for LDL calculation, as LDL‐C = (TC) − (HDL‐C) − (triglyceride[TG]/5).^1^ Hence, for most such studies, RC algebraically is simply TG/5. Direct LDL measurements can be employed to more accurately estimate RC, but, if applied to persons not having marked hypertriglyceridemia (>400 mg/dL), RC remains strongly related to TG.

Recall that TG levels below 400 mg/dL are associated with insulin resistance.^2^ Recognizing this, the myriad recent publications showing that RC correlates with CVD (cardiovascular disease),^3^ cognitive impairment,^4^ visceral adipose tissue,^5^ chronic kidney disease,^6^ “metabolically unhealthy obesity”,^7^ metabolic dysfunction‐associated steatotic liver disease,^8^ and so on appear likely to be a restatement of previously recognized associations.

Another “peeve” is the receipt of a manuscript studying an index based on the sum or ratio of several factors associated with a diabetes‐related endpoint. For example, insulin resistance is associated both with low HDL‐C and with elevations in alanine aminotransferase (ALT). If one calculates the ALT/HDL‐C ratio, then mathematically one would expect an association with insulin resistance, which might have a numerically greater correlation than with either parameter individually.^9^ However, unless the proposed new ratio allows a conceptual advance to be made, we would tend to think that little novel understanding would ensue.

Other studies have used, as a measure of insulin resistance in type 1 diabetes, the estimated glucose disposal rate, calculated as (estimated glucose disposal rate) eGDR = 24.31 − (12.22 × (waist‐hip ratio) WHR) − (3.29 × [history (high blood pressure) HBP]) − (0.57 × HbA1c). It is certainly logical that such clinical factors would correlate with the glucose disposal rate, and an insulin clamp study with a relatively small number of persons was in fact used in the derivation of this equation.^10^ It is more likely, however, that studies showing an association of this calculation of eGDR with adverse outcome,^11^, ^12^ are simply showing the effects of obesity, hypertension, and glycemic control. Similarly, we have had several recent submissions showing adverse outcome associated with the “stress hyperglycemia ratio” (the ratio of glucose to HbA1c during an acute illness), the “cardiometabolic index” (waist/height) × (TG/HDL), the “systemic inflammation response index” (neutrophil count × monocyte count/lymphocyte count). None of these seem likely to improve our true understanding of patient characteristics in such a way as to lead to better treatment approaches.

Fortunately, the many advances in the field of diabetes are ample to give us material for ongoing important discoveries, and we look forward to receiving and publishing fascinating new manuscripts pertaining to truly new understandings and treatments.
